# *Francisella tularensis* Elicits IL-10 via a PGE_2_-Inducible Factor, to Drive Macrophage MARCH1 Expression and Class II Down-Regulation

**DOI:** 10.1371/journal.pone.0037330

**Published:** 2012-05-17

**Authors:** Danielle Hunt, Justin E. Wilson, Karis A. Weih, Satoshi Ishido, Jonathan A. Harton, Paul A. Roche, James R. Drake

**Affiliations:** 1 Center for Immunology and Microbial Disease, Albany Medical College, Albany, New York, United States of America; 2 Department of Microbiology and Immunology, University of North Carolina, Chapel Hill, North Carolina, United States of America; 3 Laboratory for Infectious Immunity, RIKEN Research Center for Allergy and Immunology, RIKEN Yokohama Institute, Yokohama, Kanagawa, Japan; 4 Experimental Immunology Branch, National Cancer Institute, National Institutes of Health, Bethesda, Maryland, United States of America; Oklahoma Medical Research Foundation, United States of America

## Abstract

*Francisella tularensis* is a bacterial pathogen that uses host-derived PGE_2_ to subvert the host's adaptive immune responses in multiple ways. Francisella-induced PGE_2_ acts directly on CD4 T cells to blunt production of IFN-γ. Francisella-induced PGE_2_ can also elicit production of a >10 kDa soluble host factor termed FTMØSN (*F. tularensis* macrophage supernatant), which acts on IFN-γ pre-activated MØ to down-regulate MHC class II expression via a ubiquitin-dependent mechanism, blocking antigen presentation to CD4 T cells. Here, we report that FTMØSN-induced down-regulation of MØ class II is the result of the induction of MARCH1, and that MØ expressing MARCH1 “resistant” class II molecules are resistant to FTMØSN-induced class II down-regulation. Since PGE_2_ can induce IL-10 production and IL-10 is the only reported cytokine able to induce MARCH1 expression in monocytes and dendritic cells, these findings suggested that IL-10 is the active factor in FTMØSN. However, use of IL-10 knockout MØ established that IL-10 is *not* the active factor in FTMØSN, but rather that Francisella-elicited PGE_2_ drives production of a >10 kDa host factor *distinct from IL-10*. This factor then drives MØ IL-10 production to induce MARCH1 expression and the resultant class II down-regulation. Since many human pathogens such as *Salmonella typhi*, *Mycobacterium tuberculosis* and *Legionella pneumophila* also induce production of host PGE_2_, these results suggest that a yet-to-be-identified PGE_2_-inducible host factor capable of inducing IL-10 is central to the immune evasion mechanisms of multiple important human pathogens.

## Introduction

*F. tularensis* is a bacterial pathogen that infects macrophages and uses host-derived PGE_2_ to enhance bacterial growth and subvert the adaptive immune response [1]. Other clinically relevant human pathogens such as *Salmonella typhi* [2], [3], *Mycobacterium tuberculosis* [4] and *Legionella pneumophila* [5] also elicit host PGE_2_, and thus may employ a similar strategy to promote infection. Francisella-induced PGE_2_ acts directly on CD4 T cells, re-programming them to restrict production of anti-Francisella cytokines such as interferon-γ (IFN-γ) [1]. Moreover, blockade of Francisella-induced PGE_2_ production *in vivo* allows for more robust IFN-γ production and better control of pulmonary Francisella infection in mice [6].

**Figure 1:**
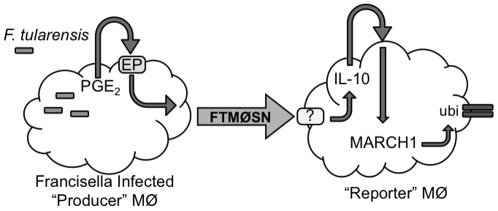
FTMØSN Production and Action. *F. tularensis* infected “producer” MØ make PGE_2_, which acts in an autocrine/paracrine fashion to drive the production of FTMØSN (*F. tularensis* MØ supernatant). FTMØSN contains a soluble factor of >10 kDa molecular mass that elicits the ubiquitin-dependent down-regulation of “reporter” MØ MHC class II molecules [7]. In this report, we establish that the active factor in FTMØSN is distinct from IL-10, but that “reporter” MØ class II down-regulation is driven by induction of *reporter* MØ IL-10 production, which drives MARCH1 expression and class II ubiquitination. This means that FTMØSN contains a factor distinct from IL-10, which is induced by *F. tularensis*-elicited PGE_2_ and which is able to drive reporter MØ IL-10 production.

Francisella-induced PGE_2_, which is elicited by either the live vaccine strain (LVS) or human virulent SchuS4 strain of Francisella, also acts in an autocrine/paracrine fashion to drive production of a soluble MØ factor that elicits the ubiquitin-dependent down-regulation of MHC class II and CD86 molecules expressed by IFN-γ activated MØ [7] (Figure 1). The factor within this *F. tularensis MØ* supernatant (termed FTMØSN, pronounced foǒt-mō-sin) drives MØ class II down-regulation by eliciting ubiquitination of the class II cytoplasmic tail, which results in class II trafficking to degradative intracellular compartments [7], [8]. The resulting class II negative MØ are thus greatly impaired in their ability to present antigens to CD4 T cells.

Until now, the identity of the active factor in FTMØSN and the mechanism of class II ubiquitination were unknown. Here, we establish that the mechanism of FTMØSN induced class II ubiquitination is via up-regulation of the ubiquitin ligase MARCH1. Studies with IL-10 knockout (IL-10Δ) MØ revealed that Francisella (via elicitation of PGE_2_) is inducing production of a yet-to-be identified factor that is able to elicit MØ IL-10 production to then induce MØ class II down-regulation via a MARCH1-dependent mechanism.

## Methods

### Ethics Statement

Mice were house and used in strict accordance to the guidelines established by the Albany Medical College Institutional Animal Care and Use Committee. Animal protocols were reviewed and approved by the Albany Medical College Institutional Animal Care and Use Committee (Protocol # 603276).

### Generation of Bone Marrow-Derived Macrophages

Producer bone marrow-derived macrophages (BMMØ – Figure 1) were generated from B10.Br, C57Bl/6 or IL-10Δ (on the C57Bl/6 background [9]) mice as previously described [7]. Reporter BMMØ from B10.Br, MARCH1Δ [10], class II K>R [11] or IL-10Δ [9] mice were treated with 100 U/ml IFN-γ for 24 hours and then washed before exposure to FTMØSN or control supernatants.

### Measurement of IL-10

IL-10 was measured by cytometric bead array (CBA, BD Biosciences, San Jose, CA catalog number 558300) IL-10 Flex Set according to the manufacturer's directions.

### Treatment of BMMØ with FTMØSN

FTMØSN was generated by exposure of producer BMMØ to *F. tularensis* LVS at an multiplicity of infection of 100∶1 (Ft∶MØ) as previously described [7]. FTMØSN was cleared of any bacteria and cellular debris by centrifugation and 0.2 µm filtration. Reporter MØ were exposed to 50% FTMØSN or control supernatants for 20–24 hours before washing and harvesting to generate whole cell lysates. The anti-IL-10 receptor blocking mAb 1B1.3A (BioXCell # BE005) was used at the indicated concentration.

### Western Blot Analysis of MØ MHC Class II Levels

Whole cell lysates of treated or control BMMØ were analyzed for total MHC class II levels by SDS-PAGE and anti-class II β chain western blot as previously reported [7]. Blots were subsequently probed for GAPDH as a loading control.

### RT-PCR Analysis of MARCH1 mRNA Levels

Total RNA was prepared using TRIzol (Invitrogen) and treated with DNase I to remove genomic DNA. RNA was converted to cDNA using standard procedures and reagents from Invitrogen. Real-time PCR primers used to amplify MHC class II and MARCH1 have been described previously [12] and primers for GAPDH were from Qiagen (QuantiTect primer #QT01658692). Real-time PCR was performed using an ABI Prism 7900HT Sequence detection system and QuantiTect SYBR green PCR kit (QIAGEN) according to the manufacturers instructions. In all experiments the normalized Ct values for the FTMØSN–treated macrophages were expressed relative to the normalized Ct values for the mock supernatant-treated macrophages.

## Results

### The Ubiquitin Ligase MARCH1 is Responsible for FTMØSN-induced Down-regulation of Macrophage Class II Expression

Previous work established that upon Francisella infection, MØ make PGE_2_, which works in an autocrine/paracrine fashion to elicit the production of a >10 kDa soluble factor termed FTMØSN. FTMØSN drives the ubiquitin-dependent down-regulation of MHC class II expressed by IFN-γ pre-activated reporter MØ (Figure 1 and [7]). The goal of this report is to define the mechanism of class II down-regulation and characterize the active factor in FTMØSN.

**Figure 2:**
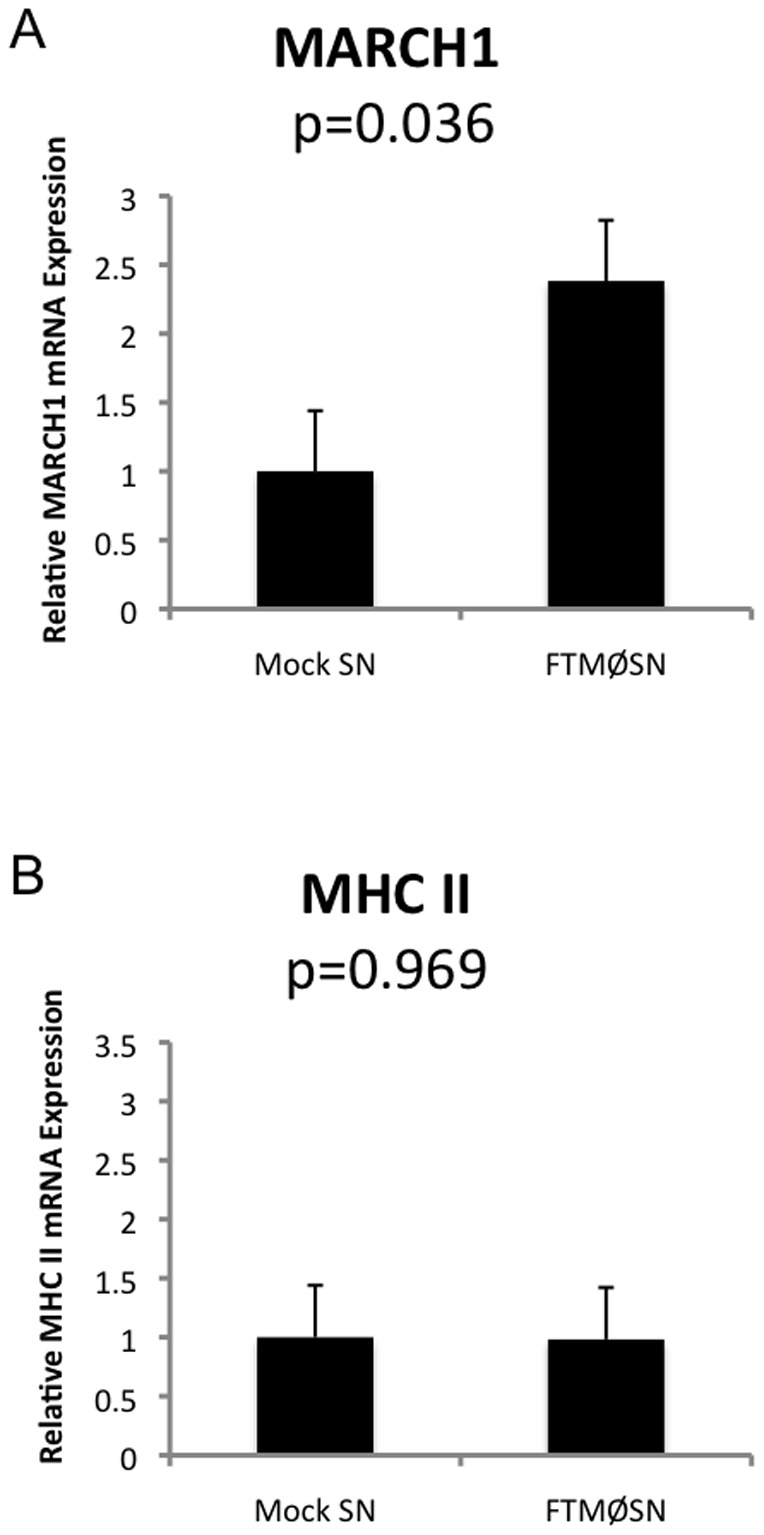
FTMØSN Induces Increased Reporter MØ MARCH1 mRNA Expression. IFN-γ pre-activated “reporter” BMMØ were treated with FTMØSN or mock SN for 24 hours. Cells were harvested and MARCH1 (panel A) or MHC class II β chain (panel B) mRNA levels determined by RT-PCR and normalized to GAPDH. Results are reported as FTMØSN-induced change in mRNA expression compared to mock SN treated MØ (+/−1 S.E.M). p values were determine by a student's *t* test. n = 3.

One mechanism for the post-translational control of MHC class II expression in MØ and dendritic cells (DC) is the regulated expression of the ubiquitin ligase MARCH1, which ubiquitinates the cytoplasmic domain of class II molecules resulting in their intracellular sorting to degradative intracellular compartments [13], [14]. To determine if MARCH1 might be involved in FTMØSN-elicited down-regulation of class II, the effect of FTMØSN treatment on reporter MØ MARCH1 mRNA levels was determined (Figure 2). Overnight treatment of IFN-γ pre-activated reporter MØ with FTMØSN results in the up-regulation of MARCH1 mRNA, but no significant change in the level of Aβ class II mRNA expression, consistent with a post-transcriptional mechanism for regulating MHC class II expression. The current lack of reliable anti-MARCH1 antibodies precludes direct analysis of the impact of FTMØSN treatment on MARCH1 protein levels. Nevertheless, the results presented in Figure 2 suggest that FTMØSN-induced up-regulation of MARCH1 may be responsible for the ubiquitin-dependent down-regulation of MHC class II molecules observed in FTMØSN treated MØ [7].

**Figure 3:**
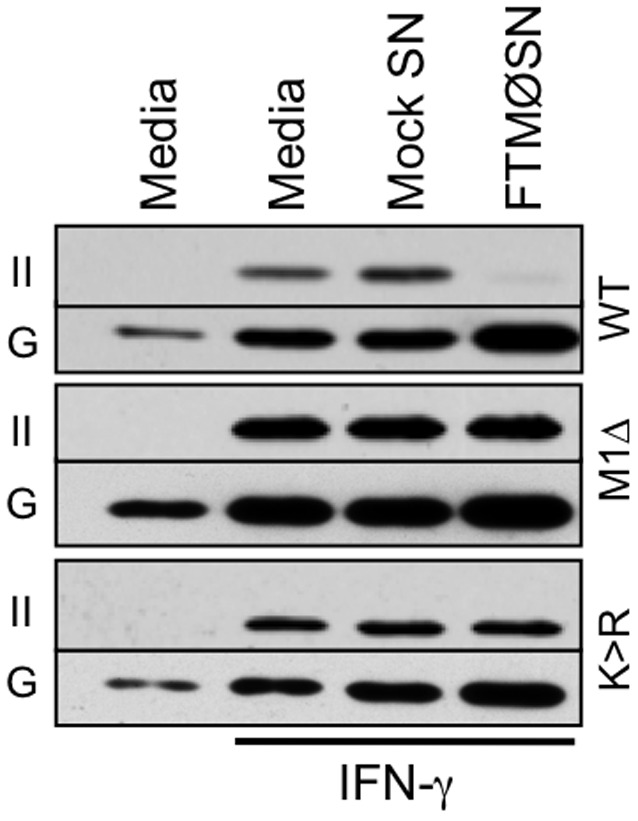
MARCH1Δ and Class II K225R BMMØ Fail to Down-Regulate Class II in Response to FTMØSN. IFN-γ activated wild type (WT), MARCH1Δ (M1Δ) or class II K225R (K>R) BMMØ were treated with mock SN or FTMØSN. After 20–24 hours, total MØ class II (II) and GAPDH (G) levels were determined by western blot analysis of whole cell lysates [7]. Shown are representative results from 1 of 3 independent experiments.

To further investigate this possibility, the level of class II expression by FTMØSN-treated wild type and MARCH1Δ reporter MØ was determined (Figure 3). The results show that MARCH1Δ MØ are completely refractory to the class II down-regulatory effects of FTMØSN, confirming the notion that FTMØSN-induced MARCH1 drives class II down-regulation. To further confirm this scenario and determine if class II is a direct target for MARCH1-mediated ubiquitination, the impact of FTMØSN treatment on class II expression by reporter MØ expressing class II molecules lacking the single cytoplasmic lysine ubiquitination site (i.e., Aβ K225R [11]) was examined (Figure 3). Consistent with the idea that class II is the direct target for MARCH1-mediated ubiquitination, MARCH1-positive MØ expressing these non-ubiquitinatable class II molecules are refractory to FTMØSN-induced MHC class II down-regulation. Taken together, the results presented in Figures 2 and 3 establish that MARCH1-mediated ubiquitination of the cytoplasmic tail of class II β chains is directly responsible for the FTMØSN-induced down-regulation of reporter MØ MHC class II expression [7].

### FTMØSN Contains a Soluble Factor the Induces MØ IL-10 Production

**Figure 4:**
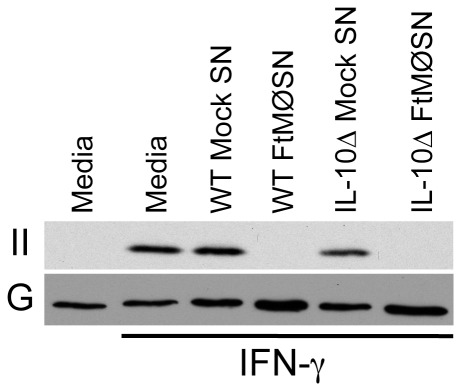
FTMØSN from IL-10 Knockout MØ Down Regulates Reporter MØ MHC Class II. Wild type (WT) and IL-10 knockout (IL-10Δ) BMMØ were treated with media or *F. tularensis* LVS to generate mock SN or FTMØSN, respectively. The resulting SN was added to IFN-γ activated B10.Br reporter MØ and after an overnight culture the levels of total MHC class II (II) and GAPDH (G) was monitored by SDS-PAGE and western blotting [7]. Shown are representative results from 1 of 3 independent experiments.

IL-10 is the only cytokine that has been demonstrated to induce MARCH1 expression in both DC and human monocytes [13], [14]. Moreover, PGE_2_ has been reported to induce MØ production of IL-10 [15] and FTMØSN contains some level of IL-10 (see below). Together, this suggests that IL-10 is likely the PGE_2_-induced factor in FTMØSN responsible for inducing MØ MARCH1 expression. To test this possibility, the ability of FTMØSN generated from both wild type and IL-10Δ “producer” MØ (Figure 1) to drive MHC class II down-regulation by wild type reporter MØ was determined (Figure 4). Surprisingly, FTMØSN from both wild type and IL-10Δ producer MØ drive the same degree of reporter MØ MHC class II down-regulation, demonstrating that the active factor in FTMØSN is not IL-10 (The absence of IL-10 in FTMØSN generated by IL10Δ BMMØ was confirmed by CBA, see below).

**Figure 5:**
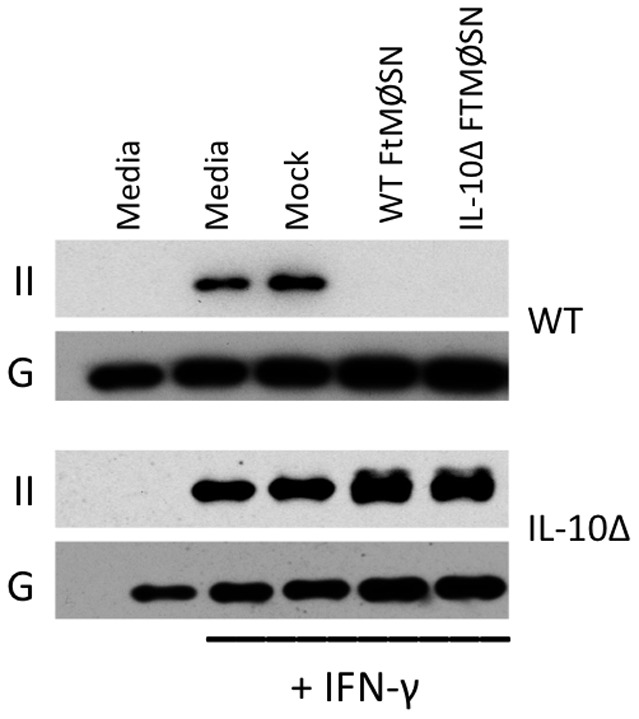
IL-10 Knockout Reporter MØ Fail to Down Regulate Class II in Response to FTMØSN. FTMØSN from wild type (WT) and IL-10Δ BMMØ was used to treat either WT (upper two panels) or IL-10Δ (lower two panels) reporter MØ. Reporter MØ were also treated with either media alone or supernatant from non-infected MØ (Mock). After overnight culture, the levels of class II (II) and GAPDH (G) was monitored as in Figure 4. Shown are representative results from 1 of 3 independent experiments.

The ability of FTMØSN from IL-10Δ producer MØ to drive MHC class II down-regulation either means that FTMØSN contains a factor distinct from IL-10 that is able to elicit MARCH1 expression and drive class II down-regulation *or* that the active factor in FTMØSN acts by inducing IL-10 production by reporter MØ which then induces MARCH1 expression and class II down-regulation. To determine which of these scenarios is correct, BMMØ from IL-10Δ mice were used as reporter MØ (Figure 5). IL-10Δ reporter MØ fail to down-regulate MHC class II expression in response to either wild type or IL-10Δ FTMØSN, whereas wild type reporter MØ down-regulate class II in response to both forms of FTMØSN. These results indicate that IL-10 produced by reporter MØ is central to FTMØSN's ability to induce class II down-regulation.

**Figure 6:**
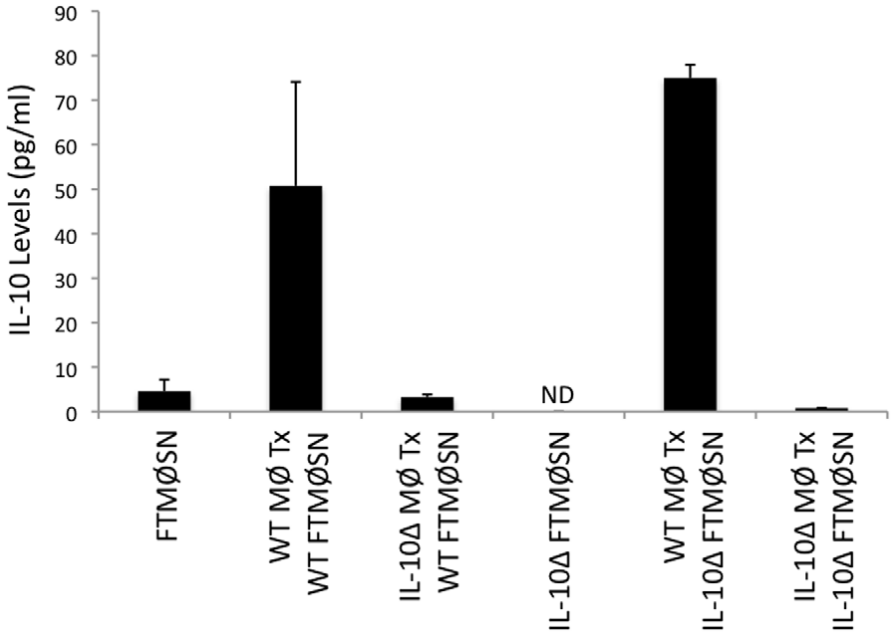
FTMØSN Induces Reporter MØ IL-10 Production. The level of IL-10 in the following samples was determined by cytometric bead array; FTMØSN from WT or IL-10Δ producer MØ (FTMØSN or IL-10Δ FTMØSN) as well as secondary SN from the indicated reporter MØ treated (Tx) with the indicated form of FTMØSN. Shown is the average level of IL-10 in each sample (+/−1 S.E.M.). n = 3 for all samples except those involving the use of IL-10Δ MØ, where n = 2 . ND = not detected. The IL-10 in the secondary SN of IL-10Δ MØ treated with WT FTMØSN is likely carry-over from the WT FTMØSN.

**Figure 7:**
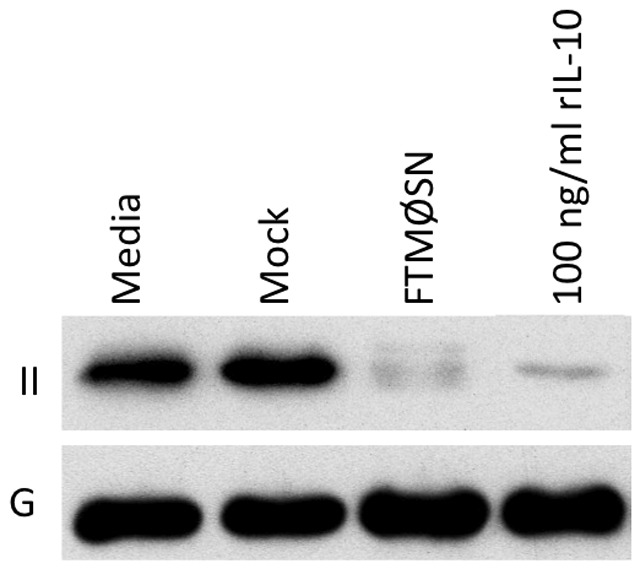
Recombinant IL-10 Down-regulates Reporter MØ Class II Expression. Reporter MØ were treated with FTMØSN or 100 ng/ml of recombinant IL-10 (rIL-10). After overnight culture, the levels of class II (II) and GAPDH (G) was monitored as in Figure 4. Shown are representative results from 1 of 3 independent experiments.

**Figure 8:**
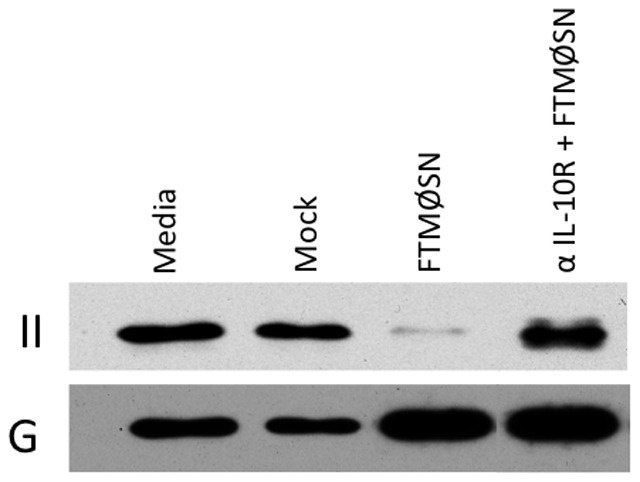
Blocking the IL-10 Receptor Blocks FTMØSN-induced Reporter MØ Class II Down-regulation. Reporter MØ were treated with 20 mg/ml anti-IL-10R blocking mAb for 20 min. at 37uC or left untreated before addition of an equal volume of undiluted FTMØSN. After overnight culture, the levels of class II (II) and GAPDH (G) was monitored as in Figure 4. Shown are representative results from 1 of 3 independent experiments.

Consistent with the possibility that FTMØSN is acting through induction of IL-10, analysis of both FTMØSN and secondary supernatants from FTMØSN-treated reporter MØ revealed that these secondary supernatants contain approximately 10 fold more IL-10 than FTMØSN itself (Figure 6). Moreover, treatment of reporter MØ with high dose recombinant IL-10 (100 ng/ml) results in down-regulation of MØ class II (Figure 7), whereas lower doses of recombinant IL-10 had a limited effect on MØ class II levels (not shown). Finally, treatment of wild type reporter MØ with a blocking anti-IL-10 receptor mAb blocks FTMØSN-induced class II down-regulation (Figure 8). Taken together, these results indicate that FTMØSN contains a PGE_2_-inducible factor *distinct from IL-10* that acts to induce IL-10 release by reporter MØ. This reporter MØ-produced IL-10 then acts in an autocrine/paracrine fashion to induce MARCH1 expression to drive ubiquitin-dependent MHC class II down-regulation.

## Discussion

Previous studies have established that *Francisella tularensis*-infected MØ produce PGE2, which can both alter CD4 T cell cytokine production and drive the production of a soluble factor (i.e., FTMØSN) that elicits the ubiquitin-dependent down-regulation of MØ MHC class II expression [7]. Since other clinically relevant human pathogens such as *Salmonella typhi* [2], [3], *Mycobacterium tuberculosis* [4] and *Legionella pneumophila* [5] also elicit host PGE2, it was of interest to determine the mechanism of FTMØSN-induced MØ class II down-regulation.

Post-translation control of MHC class II expression in DC and MØ can be driven by MARCH1-mediated ubiquitination of the cytoplasmic tail of class II molecules [13], [14], which results in altered intracellular class II trafficking away from recycling endosomes and toward degradative compartments [8]. To date, IL-10 is the only cytokine reported to induce antigen presenting cell MARCH1 expression and class II down-regulation [13], [14]. Since PGE_2_ can induce MØ IL-10 [13], we explored the possibility that a PGE_2_ to IL-10 to MARCH1 axis is responsible for the FTMØSN-induced down-regulation of reporter MØ class II expression (Figure 1).

Analysis of reporter MØ exposed to FTMØSN revealed that they up-regulate MARCH1 mRNA expression. In addition, MARCH1Δ MØ fail to down-regulate class II expression in response to FTMØSN. Finally, MØ expressing class II molecules refractory to the action of MARCH1 (i.e., I-A β chain K225R [11]) fail to down-regulate class II in response to FTMØSN. Taken together, these results establish that the active factor in FTMØSN drives the ubiquitin-dependent down-regulation of MØ class II by increasing MØ MARCH1 expression, which results in the ubiquitination of the cytoplasmic domain of class II and its post-endocytic sorting into a degradative compartment.

To address the question of whether the MARCH1-inducing factor in FTMØSN is IL-10, IL-10Δ producer MØ were exposed to *F. tularensis* and the resulting FTMØSN tested for activity. Surprisingly, FTMØSN from IL-10 knockout MØ is able to drive reporter MØ class II down-regulation, ruling out IL-10 as the active factor in FTMØSN. In contrast, IL-10Δ reporter MØ fail to down-regulate class II in response to FTMØSN (both wild type and IL-10Δ FTMØSN). These results indicate that PGE_2_ is inducing IL-10 via an indirect mechanism. More precisely, PGE_2_ is inducing the production of a >10 kDa soluble factor *distinct from IL-10* (i.e. the active factor in FTMØSN), which then acts to induce reporter MØ IL-10 production, driving MARCH1 expression and class II down-regulation. While previous reports have demonstrated that PGE_2_ can induce IL-10 production by MØ and DC [15], [16], the molecular mechanism of IL-10 induction remains undetermined. These current findings suggest a complex mechanism, minimally involving the production of at least one yet to be defined soluble factor. These results also suggest that Francisella might act to control MØ responsiveness to this undefined soluble factor, since Francisella-infected producer MØ (which are bathed in FTMØSN) do not make as much IL-10 at non-infected reporter MØ exposed to FTMØSN. This possibility will be addressed in future studies.

Presently, the identity of the IL-10-inducing active factor in FTMØSN remains unclear. Preliminary experiments using either knockout MØ and/or neutralizing mAbs have ruled out TGF-β, IL-6, VEGF, MIP-1α and leukemia inhibitory factor. Moreover, generation of supernatants from Francisella–infected gene knockout producer MØ has established that production of FTMØSN activity is *in*dependent of TLR-2 (which recognizes *F. tularensis*-derived lipoproteins [17]) and MØ caspase-1 activity (which drives IL-1β production/release). Therefore, it is likely that a combined genomics/proteomics approach will be necessary to conclusively identify and characterize the PGE_2_-induced active factor in FTMØSN that elicits MØ IL-10 expression to then drive MØ class II down-regulation.

MHC class II restricted antigen presentation by MØ is central to immune defense against many human pathogens and tumors. Moreover, many human pathogens and tumors elicit the production of significant levels of PGE_2_. The results presented in this report establish that PGE_2_ drives the production of a soluble MØ factor *distinct from IL-10* that induces MØ IL-10 production to drive MARCH1 expression, culminating in down-regulation of MØ class II molecules and limiting the ability of these cells to participate in an adaptive immune response. Future identification of the active factor in FTMØSN will provide greater insight into this mechanism of immune suppression common to both infectious agents and tumors, and will provide an additional target for new interventional therapies.
